# Effects of Diet Composition on Postprandial Energy Availability during Weight Loss Maintenance

**DOI:** 10.1371/journal.pone.0058172

**Published:** 2013-03-06

**Authors:** Carolyn O. Walsh, Cara B. Ebbeling, Janis F. Swain, Robert L. Markowitz, Henry A. Feldman, David S. Ludwig

**Affiliations:** 1 New Balance Foundation Obesity Prevention Center at Boston Children’s Hospital, Boston, Massachusetts, United States of America; 2 Center for Clinical Investigation, Brigham and Women’s Hospital, Boston, Massachusetts, United States of America; 3 Clinical Research Center, Boston Children’s Hospital, Boston, Massachusetts, United States of America; NIDDK/NIH, United States of America

## Abstract

**Background:**

The major circulating metabolic fuels regulate hunger, and each is affected by dietary composition. An integrated measure of postprandial energy availability from circulating metabolic fuels may help inform dietary recommendations for weight maintenance after weight loss.

**Aim:**

We examined the effect of low-fat (LF, 60% of energy from carbohydrate, 20% fat, 20% protein), low-glycemic index (LGI, 40%–40%-20%), and very low-carbohydrate (VLC, 10%–60%-30%) diets on total postprandial metabolic fuel energy availability (EA) during weight loss maintenance.

**Methods:**

Eight obese young adults were fed a standard hypocaloric diet to produce 10–15% weight loss. They were then provided isocaloric LF, LGI, and VLC diets in a randomized crossover design, each for a 4-week period of weight loss maintenance. At the end of each dietary period, a test meal representing the respective diet was provided, and blood samples were obtained every 30 minutes for 5 hours. The primary outcome was EA, defined as the combined energy density (circulating level×relative energy content) of glucose, free fatty acids, and β-hydroxybutyrate. Secondary outcomes were individual metabolic fuels, metabolic rate, insulin, glucagon, cortisol, epinephrine, and hunger ratings. Respiratory quotient was a process measure. Data were analyzed by repeated-measures analysis of variance, with outcomes compared in the early (30 to 150 min) and late (180 to 300 min) postprandial periods.

**Results:**

EA did not differ between the test meals during the early postprandial period (p = 0.99). However, EA in the late postprandial period was significantly lower after the LF test meal than the LGI (p<0.0001) and VLC (p<0.0001) test meals. Metabolic rate also differed in the late postprandial period (p = 0.0074), with higher values on the VLC than LF (p = 0.0064) and LGI (p = 0.0066) diets.

**Conclusion:**

These findings suggest that an LF diet may adversely affect postprandial EA and risk for weight regain during weight loss maintenance.

**Trial Registration:**

ClinicalTrials.gov NCT00315354

## Introduction

Circulating levels of the major fuels the body uses for metabolic processes, including glucose, free fatty acids (FFA), and ketones, are tightly regulated by hormonal mechanisms. When circulating levels are high, insulin promotes deposition of glucose and fatty acids into muscle, liver and adipose and suppresses their production and release from storage sites. Conversely, when circulating metabolic fuels are low, counter-regulatory hormones (especially glucagon, and also cortisol, epinephrine and growth hormone) stimulate lipolysis, glycogenolysis, gluconeogensis, and ketone formation [1].

Many studies have shown that circulating levels of the individual fuels affect appetite [2], [3], [4], [5]. In both rodent [2], [3] and human [4] models, hypoglycemia and decreased FFA levels lead to increased food intake. Limited data also suggest that ketones, such as beta-hydroxybutyrate (BHB), decrease appetite [5]. However, these studies of individual fuels may not provide a comprehensive view of the metabolic regulation of hunger, because the body can utilize a varying mix of fuels under varying dietary conditions. Indeed, numerous popular weight loss diets have advocated specific macronutrient prescriptions, in part because of their intrinsic effects on metabolism and hunger [6], [7], [8], [9].

We previously proposed that high glycemic load diets reduce availability of metabolic fuels in the postprandial period by eliciting a high insulin to glucagon ratio, leading to excessive hunger and overeating [4]. (Whereas glycemic index measures how a food or meal affects postprandial blood glucose, controlling for carbohydrate amount, glycemic load is the multiplicative product of glycemic index and carbohydrate amount [10].) The purpose of this study is to examine a novel measure of total circulating energy availability (EA), defined as the combined relative energy density (circulating level×relative energy content) of each of the major metabolic fuels, in overweight and obese young adults consuming diets ranging widely in macronutrients. Specifically, we propose that EA will be lower after a high glycemic load (low-fat, LF) diet in the postprandial period, compared to diets with a moderate glycemic load (moderate fat, low-glycemic index, LGI) or low glycemic load (very low-carbohydrate, VLC).

## Materials and Methods

### Overview

This postprandial study was conducted in the setting of a larger clinical trial, comprising run-in and test phases [11]. During the run-in phase, we collected baseline data, restricted energy intake using a balanced hypocaloric diet [12] to achieve a 10–15% loss in body weight, and determined energy requirements for maintaining weight loss. During the test phase, we conducted a three-way crossover study to evaluate isocaloric diets (LF, LGI, and VLC) in random order, under conditions of weight-loss maintenance. Postprandial outcomes were assessed following test meals, reflecting respective diets, during an inpatient hospital admission at the end of each 4-week diet. Data for this substudy were collected at Boston Children’s Hospital and Brigham and Women’s Hospital in Boston, Massachusetts between June 2006 and November 2008.

### Participants

Participants were recruited from the greater Boston area using flyers, newspaper advertisements, and Internet postings that described the study as an opportunity for weight loss with provision of meals. Inclusion criteria were: 18–40 years of age, body mass index ≥27 kg/m^2^, medical clearance from a primary care provider, and willingness to eat and drink only the foods and beverages on the study menu for the duration of the study. Exclusion criteria included: weight >160 kg, change in body weight greater than ±10% over preceding year, use of medications that might affect study outcomes, current smoking, diabetes mellitus (fasting plasma glucose level ≥126 mg/dl), or other major illness as assessed by a medical history and laboratory screening tests (thyroid stimulating hormone, complete blood count, blood urea nitrogen, creatinine, and alanine aminotransferase). For females, additional exclusion criteria included irregular menstrual cycles, pregnancy or lactation during the 12 months prior to enrollment, and change in birth control medication in the three months prior to enrollment. Participants received $500 at the end of the run-in phase and $2000 at the end of the test phase. This report is based on the first 8 participants to complete the larger clinical trial [11], for whom we obtained data with regard to all circulating metabolic fuels during the postprandial periods following test meals.

### Ethics

The study was approved by the Institutional Review Boards at Boston Children’s Hospital and Brigham and Women’s Hospital. Written informed consent was obtained from each participant. The study was registered at ClinicalTrials.gov, with identifier NCT00315354.

### Dietary Interventions

Energy needs were estimated using the Mifflin-St Jeor equation [13], [14] and an activity factor derived from a modified Seven Day Activity Questionnaire [15].

Standardized test meals are described in Table 1, and the compositions of the test meals are shown in Table 2.

**Table 1:** Standardized test meals for a 2000 kcal/day diet.

LF	LGI	VLC^1^
Instant oatmeal 53 g	Steel cut oats 45 g	Egg, sausage, and cheese bake:
Turkey sausage 40 g	Egg, whole raw 70 g	Egg, whole raw 110 g
Promise margarine 8 g	Cottage cheese, 1% 90 g	Egg whites 50 g
Milk, nonfat 205 g	Promise margarine 22 g	Pork sausage 80 g
Water, from tap 227 g	Water, from tap 236 g	Cream 20 g
Grape juice 77 g	Pink grapefruit, fresh 75 g	Light shredded cheddar cheese 20 g
Raisins 15 g	Fructose sweetener 10 g	Orange, fresh 70 g
Sugar 6 g		

All food was weighed prior to cooking.

1 A pork-free VLC option with the same macronutrient content was available for participants with religious restrictions on pork consumption.

LF = low-fat, LGI = low-glycemic index, VLC = very low-carbohydrate.

**Table 2:** Composition of standardized test meals for a 2000 kcal/day diet.

	LF	LGI	VLC^1^
Energy (kcal)	500	501	497
Carbohydrate (%)	59.9	39.7	10.2
Glycemic Index	65.2	40.5	48.0
Glycemic Load	44.0	16.7	3.1
Protein (%)	20.1	19.6	29.8
Fat-total (%)	20	40.7	60
Fat-Saturated (%)^2^	3.7	10	22.3
Fat-Monounsaturated (%)	5.3	13.6	23.8
Fat- Polyunsaturated (%)	6.5	15.4	8.9
Fat- trans (%)	0.5	0.4	0.1
Fat- other (%)	4.1	1.2	4.9
Cholesterol (mg)	41	304	545
Dietary Fiber (g)	5.8	5.3	1.7

1 A pork-free VLC option with the same macronutrient content was available for participants with religious restrictions on pork consumption.

2 Fat percentages refer to percent of total kcal from that fat source.

LF = low-fat, LGI = low-glycemic index, VLC = very low-carbohydrate.

Nutrient composition of the test meals was calculated using Food Processor Software (Food Processor SQL; ESHA Research; Salem, OR, version 9.8). The glycemic index values for carbohydrate-containing foods were assigned using published values with a glucose reference, and overall glycemic load for the meal was calculated using the following equation:
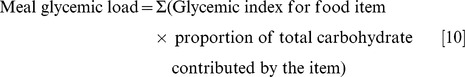



Study staff and laboratory technicians who collected outcome data were masked to diet order.

### Inpatient Hospital Admissions

Each participant underwent inpatient postprandial testing at the end of the fourth week on the three respective isocaloric diets. Participants were admitted at 5 pm the night before testing, and a nurse placed an intravenous line for blood sampling. The following morning, participants were awakened at 6∶30 am following a 10-hour fast for measurement of resting energy expenditure and a baseline blood draw. Participants then ate a breakfast, containing 25% of estimated daily energy needs and reflecting the diet composition of the respective test diet, within 15 minutes. Every 30 minutes for 5 hours, blood was drawn and participants rated hunger on a 10 cm visual analogue scale, using the prompt: “How hungry are you right now?” (with verbal anchors ranging from “Not at all hungry” to “Extremely hungry”). Metabolic rate was assessed at regular intervals throughout the postprandial period as described below. Admissions for female participants occurred during the follicular phase of the menstrual cycle to minimize potential confounding of metabolic outcomes.

### Outcome Measures

The primary outcome, total EA from metabolic fuels, was calculated as the sum, in kcal/L, of energy from glucose, FFA, and BHB, measured using standard laboratory methodology:
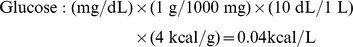


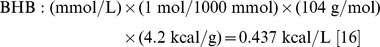


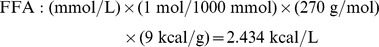



Several assumptions were made in the calculation of EA. Consistent with previous studies [17], [18], [19], BHB was used as a proxy for total ketone levels because acetoacetate, the other bioavailable ketone, is unstable and must be measured immediately after sample collection [20]. There are many different forms of FFA with different chain lengths and molecular weights; our conversion of FFA from mol/L to kcal/L was based on an estimated average chain length of 17 in light of prior studies indicating a range of 16.0–17.7 (32–36). Substituting estimated average chain lengths of 16 and 18 did not materially change the results. Additionally, the FFA calculation assumes the same energy density as triglycerides, thus ignoring the relatively minor contribution of the glycerol moiety. In calculating EA, we included only substrates that are readily available for cellular metabolism. Potential fuel sources requiring metabolic transformation prior to oxidation, such as esterified fatty acids and amino acids (which can serve as substrate for gluconeogeneis), were not included, since their metabolically available forms would be represented in our measurements of free fatty acids and glucose, respectively.

Metabolic rate was measured by indirect calorimetry at rest and then during the postprandial period using a dilution canopy system (Vmax Encore 29 N; VIASYS Healthcare Inc.; Yorba Linda, California). REE was measured while the subject was lying awake and still in a temperature-regulated room with minimal light and noise. During the postprandial period, a DVD with calm programming (i.e., travelogue) was shown to prevent boredom and sleep. At rest and following the test meal, oxygen consumption and carbon dioxide production were measured for 30 minutes of every hour. Using data averaged over the last 20 minutes of each measurement interval, energy expenditure was calculated using the Weir equation [21], and respiratory quotient was estimated as the ratio of carbon dioxide production to oxygen consumption. We assumed no change in body composition during weight stability for 1 month on each of the three diets, an assumption supported by a 6-month weight loss study showing similar loss of lean relative to fat tissue with a LF or VLC diet [22].

Secondary outcomes were glucose, FFA, BHB, glucagon, insulin, cortisol, epinephrine, metabolic rate, and hunger ratings. Respiratory quotient was included as a process measure, with possible values generally ranging from 0.7 (total fat oxidation) to 1.0 (total carbohydrate oxidation).

### Power Calculations

Assuming 80% power and a Bonferroni-corrected Type I error rate 0.05/3, the detectable pairwise difference between diets with 8 participants was calculated to be between 0.89 and 1.55 standard deviations, based on whether the intrasubject correlation was strong or weak. Post-hoc power analysis indicated that a new, identically designed crossover study with 8 participants would have more than 98% power to detect differences of the observed magnitude between the LF and other two diets.

### Statistical Analysis

In light of prior work suggesting differing patterns of EA in the early versus late postprandial period [4], we identified two time periods of interest in which to compare diets, namely 30–150 min and 180–300 min, each comprising 2 hours (5 time points). We modeled EA as a function of diet and time, with an interaction term to allow the time course to vary by diet, using repeated-measures analysis of variance with an autoregressive covariance structure to account for within-subject correlation. For each period, using parameters of the fitted model, we estimated mean EA for each diet; constructed pairwise differences among diets; and tested the hypothesis that mean EA was equal in all 3 diets. When that hypothesis was rejected at p<0.05, we performed additional pairwise comparisons with a Bonferroni-adjusted critical p-value of 0.05/3 = 0.017. Secondary outcomes, including areas under the curve, were analyzed similarly. We did not adjust for diet order, given that this 6-level covariate would consume most of our inter-subject degrees of freedom, making inferences impossible. SAS software (version 9.2, Cary, NC) was used for all computations.

## Results

### Participant Characteristics

The baseline characteristics of the eight participants are shown in Table 3. A 3-day average of body weight at the end of each diet indicated weight stability, with mean within subject variability less than 1 kg (p = 0.41).

**Table 3:** Baseline characteristics of the study population.

		Mean ± SD
Age (years)		30.8±6.4
Body Mass Index (kg/m^2^)	Pre-weight loss	33.4±4.8
	Post-weight loss	29.4±4.0
Body fat (%)	Pre-weight loss	33.5±7.9
	Post-weight loss	29.6±9.1
Blood pressure (mmHg)	Systolic	115±14.2
	Diastolic	70±7.3
Fasting blood glucose (mg/dL)		91.2±10.6
Fasting lipids (mg/dL)	Total cholesterol	177±34.9
	Triglycerides	154±97.7
	HDL cholesterol	47.5±11.0
	LDL cholesterol	98.4±24.3
		**n (%)**
Sex	Male	4 (50)
	Female	4 (50)
Race	Black	3 (37.5)
	White	1 (12.5)
	Asian	2 (25)
	Other (Caribbean)	1 (12.5)
	No response	1 (12.5)
Ethnicity	Hispanic	1 (12.5)
	Not Hispanic	7 (87.5)

Unless otherwise specified, data are from pre-weight loss baseline testing. Body fat percent was measured by dual-X ray absorptiometry.

### Primary Outcome- Energy Availability

**Figure 1:**
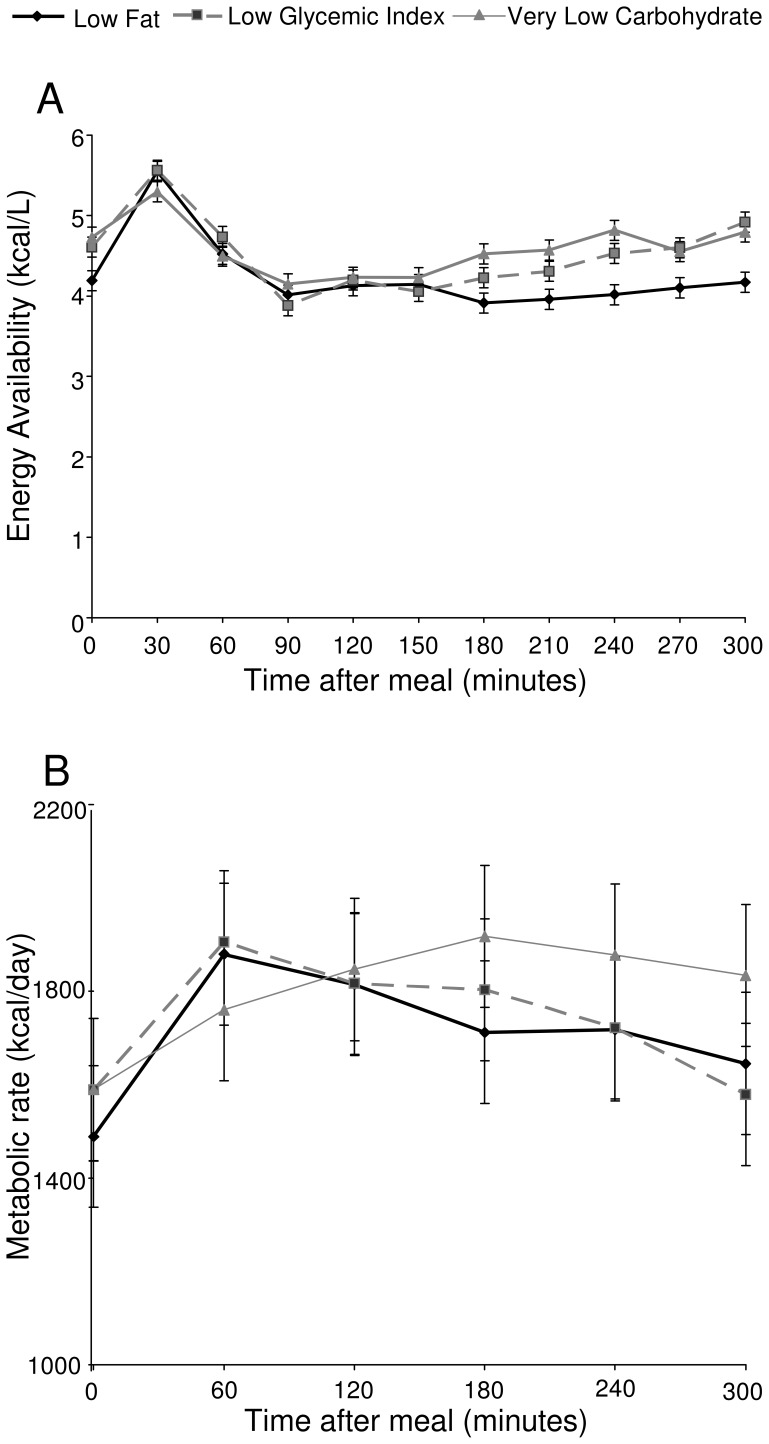
Postprandial energy availability (EA) (Panel A, kcal/L), and metabolic rate (Panel B, kcal/day). EA is calculated as the total energy densities of glucose, free fatty acids, and β-hydroxybutyrate. Error bars represent the standard error of the mean from fitted repeated-measures model.

Postprandial EA is shown in Figure 1, panel A. At baseline, prior to the test meals, EA was significantly different among the three groups (p = 0.0024), due to lower levels with the LF diet compared with either the LGI (p = 0.0096) or VLC (p = 0.0009) diet. In the early postprandial period 30–150 minutes after the test meal, EA did not differ significantly among diets (p = 0.99). However, in the late postprandial period 180–300 minutes after respective test meals, EA differed significantly among diets (p<0.0001), due to lower levels with the LF diet compared with either the LGI (p<0.0001) or VLC (p<0.0001) diet. Mean EA in the late postprandial period was 4.03 kcal/L with the LF diet, compared with 4.52 kcal/L with the LGI diet and 4.65 kcal/L with the VLC diet. An analysis of the area under the curve for each diet in the early and late postprandial period showed similar results, with no difference between the diets in the early postprandial period (p = 0.8), and a significant difference in the late postprandial period (p = 0.008), with lower area under the curve with the LF diet than both LGI (p = 0.004) and VLC (p = 0.0003) diets, and no difference between LGI and VLC diets (p = 0.2).

### Metabolic Rate

Metabolic rate is shown in Figure 1, panel B. Metabolic rate did not differ by diet in the early postprandial period (p = 0.67). However, in the late postprandial period, metabolic rate was higher after the VLC diet (p = 0.0074) than the LF (p = 0.0064) or LGI (p = 0.0066) diet.

#### Respiratory quotient

Respiratory quotient values ranged from 0.88–0.94 after the LF meal, 0.80–0.88 after the LGI meal, and 0.77–0.80 after the VLC meal. All pairwise comparisons for respiratory quotient indicated significant differences over the entire curve, with respiratory quotient higher with the LF diet vs. the LGI (p<0.0001) or VLC (p<0.0001) diet, and with the LGI vs. VLC diet (p = 0.0002).

#### Metabolic fuels

**Figure 2:**
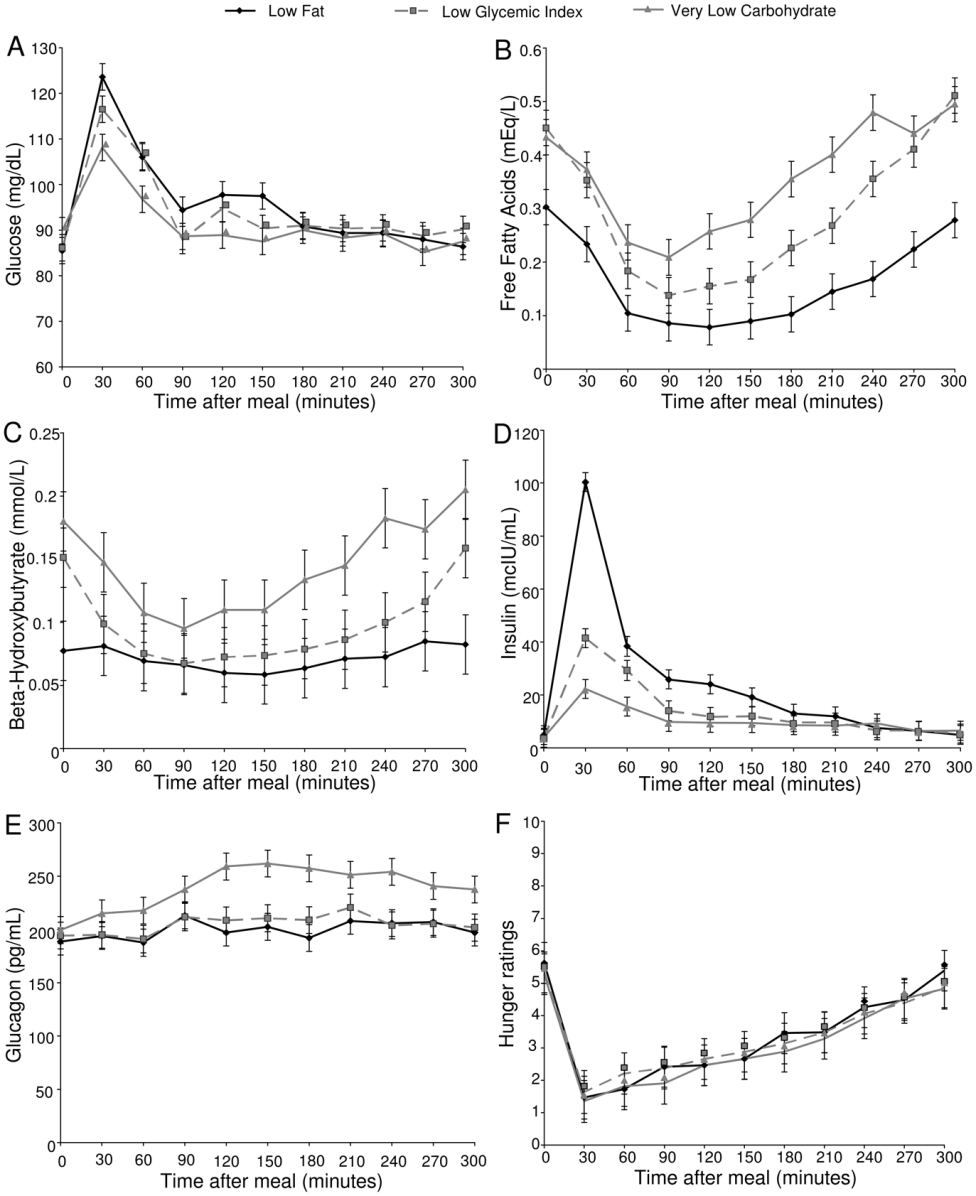
Postprandial levels of metabolic fuels, hormones, and hunger. The figures show levels of glucose (Panel A, mg/dL), free fatty acids (Panel B, mEq/L), β-hydroxybutyrate (Panel C, mmol/L), insulin (Panel D, mcIU/mL), and glucagon (Panel E, pg/mL), and hunger ratings (Panel F). Error bars represent the standard error of the mean from fitted repeated-measures model.

Circulating levels of individual metabolic fuels are shown in Figure 2.

For glucose, there was a significant effect of diet (p = 0.036), as well as a diet×time interaction (p = 0.0001). In the early postprandial period the three diets differed significantly (p = 0.0002), with glucose higher with the LF diet than the VLC diet (p<0.0001). The other two comparisons fell short of the Bonferroni criterion for significance, with glucose showing a higher response to the LF diet vs. the LGI diet (p = 0.048) and the LGI diet vs. the VLC diet (p = 0.031). There was no effect of diet on the level of the glucose curves in the late postprandial period (p = 0.66).

With both FFA and BHB, there was a significant effect of diet over the whole curve (FFA p<0.0001, BHB p = 0.024), and a diet×time interaction (FFA p = 0.0002, BHB p<0.0001). Over the whole curve, the FFA level was lower with the LF diet than with the LGI (p<0.0001) and VLC (p<0.0001) diets, and lower by a marginally non-significant amount with the LGI versus the VLC diet (p = 0.018). The BHB level over the whole curve was lower with the LF diet compared with the VLC diet (p = 0.007).

The FFA:BHB ratio differed between diets. At baseline (t = 0), the ratio differed between diets (p = 0.02); it was higher with the LF diet than with the LGI diet (p = 0.015), and higher with the LF than with VLC diet, with borderline significance (p = 0.02). The ratio also differed over the entire postprandial curve (p = 0.02) in the other direction; the ratio was lower with the LF diet than with LGI diet (p = 0.007), and lower with the LF diet than with the VLC diet with borderline significance (p = 0.04).

#### Hormones

Postprandial insulin and glucagon are shown in Figure 2. For both of these hormones, there were significant effects of diet (insulin p<0.0001, glucagon p<0.0001) as well as diet×time interactions (insulin p<0.0001, glucagon p = 0.045). Insulin level was affected by diet in the early postprandial period (p<0.0001), being higher with the LF diet than the LGI (p<0.0001) and VLC (p<0.0001) diets, and higher with the LGI diet than the VLC diet (p = 0.0001). There was no such effect in the late postprandial period (p = 0.84). Over the entire curve, glucagon was higher with the VLC diet than with the LF (p<0.0001) and LGI (p = 0.0001) diets.

For cortisol, there was a significant effect of time (p<0.0001) over the entire period, but no effect of diet (p = 0.37) and no diet×time interaction (p = 0.72). For epinephrine, there was no significant effect of time (p = 0.27) or diet (p = 0.20), and no diet×time interaction (p = 0.39).

#### Hunger ratings

Hunger ratings are shown in Figure 2. There was a significant effect of time on hunger ratings (p<0.0001), but no effect of diet (p = 0.89) and no diet×time interaction (p = 0.91). Hunger did not differ significantly among diets in either the early (p = 0.87) or late (p = 0.86) postprandial period.

## Discussion

In this study, we examined the postprandial effects of three common dietary patterns in young adults during a period of weight maintenance after weight loss. We proposed a novel outcome measure, EA, which combines the relative energy available from three major metabolic fuels (glucose, FFA, and BHB) and hypothesized that an LF meal would lower total metabolic fuel availability and energy expenditure in the late postprandial period by causing a high insulin to glucagon ratio secondary to a high dietary glycemic load.

As hypothesized, the LF meal led to lower EA in the late postprandial period compared with the LGI and VLC meals. Consistent with previous studies [4], [23], [24], [25], the early postprandial period after an LF meal was characterized by a high glucose level and concomitant exaggerated insulin response. Despite the high glucose peak 30 minutes after the LF meal, EA did not differ in the early postprandial period due to suppression of FFA and BHB after this meal. In the late postprandial period, the expected reactive hypoglycemia [23], [24], [25] did not occur; differences in late postprandial EA were instead due to significantly lower levels of FFA after the LF meal as compared with the LGI and VLC meals, and lower levels of BHB after the LF meal as compared with the VLC meal. These patterns are similar to those observed with testing after a single meal, suggesting that the difference in EA is likely not due to habituation to a given dietary pattern [4].

Similarly to EA, metabolic rate did not differ after the three test meals in the early postprandial period but differed significantly in the late postprandial period, with higher metabolic rate after a VLC meal than an LF or LGI meal. This sustained high postprandial metabolic rate after the VLC meal may contribute to the findings that total energy expenditure decreases less after weight loss on a VLC diet than on an LF or LGI diet [11], with potential implications for risk of weight regain.

There was no significant effect of diet on hunger ratings. Unlike prior work examining the effect of similar test meals on postprandial hunger [4], this study relied on hunger ratings and did not measure *ad libitum* food intake elicited by hunger, a potentially more valid assessment. Several studies have shown that hunger ratings do not necessarily predict equal weight regain on the three diets [26], [27]. Moreover, our methods may not adequately distinguish between homeostatic hunger, which develops over hours in the absence of caloric intake, and hedonic hunger, which relates to the presence of palatable food and environmental eating cues [28].

A strength of the current study is the novelty of the primary outcome, a composite measure of EA comprising the relative availability of energy from glucose, FFA, and ketones. In addition, the protocol was conducted in a highly controlled setting allowing for accurate formulation of the test diets and collection of outcome data. Furthermore, the significant separation of respiratory quotient values, and the differences in glucose and insulin as expected based on calculated dietary glycemic load, show that we achieved the desired physiologic differentiation between diets. For these reasons, we can confidently assume that the data reflect the physiologic effect of the planned dietary interventions. Other strengths include the use of a crossover design in which each participant served as his or her own control, thereby limiting variability and mitigating the effect of the small sample size, and the selection of diets that span the full range of prevailing macronutrient compositions.

This study also has several limitations. Despite the strength of crossover design and the high power, the sample size was small, limiting generalizability. Several assumptions were made in calculating EA, though these assumptions rest upon physiological principles and withstand sensitivity analysis (in the case of FFA). The analysis did not include lactate, a labile metabolite that may be higher after an LF meal than a VLC meal [29]. For this reason, failure to account for lactate may produce bias favoring the study hypothesis. However, the difference in lactate concentration between LF and VLC meals in a previous study was only about 0.2 mmol/L throughout the late postprandial period [29]. Using a molecular weight of 90 and an energy content of 3.6 kcal/g, a 0.2 mmol/L difference in lactate concentration would equal 0.06 kcal/L, a relatively small effect compared with the observed difference of 0.62 kcal/L between the LF and VLC diets in the late postprandial period in our study. Moreover, the use of BHB as a proxy for total ketones, without also taking acetoacetate into account, would underestimate EA in dietary conditions with higher total ketone concentration, such as the VLC diet, thereby producing bias toward the null hypothesis. Because the normal ratio of BHB to acetoacetate is 1∶1 [30], we would expect a mean difference in acetoacetate concentration between the LF and VLC meals in the late postprandial period to equal about 0.1 mmol/L, adding 0.05 kcal/L to the difference between these two meals during that time period (using a molecular weight of 102, and an energy content of 5 kcal/g). Therefore, the unaccounted EA related to acetoacetate essentially counterbalances any bias related to lactate. Another concern is that our measure of EA is based upon absolute concentrations of metabolic fuels, rather than flux into and out of cells. Additional research will be needed to determine how dietary composition may affect these fluxes. Nevertheless, the absolute concentration of nutrients has inherent relevance, as implicitly recognized by the concept of hypoglycemia [4]. Moreover, we cannot ascribe findings from this study to the effects of any specific macronutrient, as the diets differed in many ways from one another. However, the similarity in protein content between the LF and LGI diets rules out a primary role of this key macronutrient. A final limitation arises from the lack of data on *ad libitum* energy intake in the postprandial period.

In conclusion, this study finds that EA in the postprandial state differs among diets, and may have implications for weight maintenance after weight loss. This finding does not support the use of LF diets, as presently endorsed by many organizations [6], [7], [31], [32], for weight-loss maintenance. Additional research in this area appears to be warranted, including more careful assessment of homeostatic and hedonic hunger and comparison of EA and rates of weight regain.
